# Proteomic Profiling of Mouse Liver following Acute *Toxoplasma gondii* Infection

**DOI:** 10.1371/journal.pone.0152022

**Published:** 2016-03-22

**Authors:** Jun-Jun He, Jun Ma, Hany M. Elsheikha, Hui-Qun Song, Dong-Hui Zhou, Xing-Quan Zhu

**Affiliations:** 1 State Key Laboratory of Veterinary Etiological Biology, Key Laboratory of Veterinary Parasitology of Gansu Province, Lanzhou Veterinary Research Institute, Chinese Academy of Agricultural Sciences, Lanzhou, Gansu Province 730046, PR China; 2 College of Veterinary Medicine, Hunan Agricultural University, Changsha, Hunan Province 410128, PR China; 3 Faculty of Medicine and Health Sciences, School of Veterinary Medicine and Science, University of Nottingham, Sutton Bonington Campus, Loughborough, LE12 5RD, United Kingdom; University of Cambridge, UNITED KINGDOM

## Abstract

*Toxoplasma gondii* remains a global public health problem. However, its pathophysiology is still not-completely understood particularly the impact of infection on host liver metabolism. We performed iTRAQ-based proteomic analysis to evaluate early liver protein responses in BALB/c mice following infection with *T*. *gondii* PYS strain (genotype ToxoDB#9) infection. Our data revealed modification of protein expression in key metabolic pathways, as indicated by the upregulation of immune response and downregulation of mitochondrial respiratory chain, and the metabolism of fatty acids, lipids and xenobiotics. *T*. *gondii* seems to hijack host PPAR signaling pathway to downregulate the metabolism of fatty acids, lipids and energy in the liver. The metabolism of over 400 substances was affected by the downregulation of genes involved in xenobiotic metabolism. The top 10 transcription factors used by upregulated genes were Stat2, Stat1, Irf2, Irf1, Sp2, Egr1, Stat3, Klf4, Elf1 and Gabpa, while the top 10 transcription factors of downregulated genes were Hnf4A, Ewsr1, Fli1, Hnf4g, Nr2f1, Pparg, Rxra, Hnf1A, Foxa1 and Foxo1. These findings indicate global reprogramming of the metabolism of the mouse liver after acute *T*. *gondii* infection. Functional characterization of the altered proteins may enhance understanding of the host responses to *T*. *gondii* infection and lead to the identification of new therapeutic targets.

## Introduction

Over one-third of human population worldwide are chronically infected with the apicomplexan protozoan parasite *Toxoplasma gondii* [1], and are thus at high risk of developing serious diseases if they became immune-compromised or when women get pregnant. Despite great effort and much progress, toxoplasmosis remains a major threat to global health. No vaccine is available to date; anti-toxoplasma drugs are not highly effective and are associated with significant side effects [2]. Also, the parasite is capable of developing resistance to these drugs. Evidently, the current toxoplasmosis treatments have limitations. Novel approaches and new therapeutic targets need to be explored. The wide range of susceptible hosts and the remarkable ability of *T*. *gondii* to infect and replicate within almost any nucleated cell [1], resulted in *T*. *gondii* being associated with a variety of clinical disorders. However, brain and spinal cord have been always the primary targets of *T*. *gondii* infection.

Interestingly, many studies have also indicated that the liver is another target organ for *T*. *gondii* replication, as evidenced by the several hepatic pathologies, such as hepatomegaly, hepatitis, granuloma [3], necrosis, cholestatic jaundice [4], and cirrhosis [5,6], associated with *T*. *gondii* infection. Also, *T*. *gondii* infection has been incriminated in causing abnormal liver function tests in mice, including alanine aminotransferase (ALT) and aspartate aminotransferase (AST) six days post-infection in mice [7] and liver dysfunction in liver and kidney of transplant recipients [8]. Acute *T*. *gondii* infection in mice with RH strain revealed an association between the increased number of hepatic stellate cells (HSCs) and the amount of *T*. *gondii* antigens, suggesting a modulatory role for HSCs in the pathogenesis of *T*. *gondii*-induced hepatopathy [9]. Furthermore, *T*. *gondii* infection can induce pharmacokinetic changes [10,11]. These data indicate that hepatic toxoplasmosis does exist, but probably because *T*. *gondii* spreads to the liver during the acute phase of infection it may go unnoticed.

Although it is generally known that parasitic and host factors contribute to the pathogenesis and progression of toxoplasmosis, the interplay between *T*. *gondii* and host liver remains poorly understood. This is surprising giving the fact that liver is an essential organ that can carry out a wide variety of critical operations, such as detoxification of xenobiotics and producing bile that facilitates digestion. Functional change in the liver could result in severe disease, such as cancer, malnutrition, hepatitis, jaundice, cirrhosis, and adverse drug reactions [12]. Different tools have been used to investigate molecular mechanisms of toxoplasmosis, including proteomics [13–15], transcriptomics [16,17], and metabolomics [18,19]. Specifically, proteomics can provide high-throughput methods such that hundreds of proteins can be identified and/or quantified in a single analysis.

Mass spectrometry-based proteomic approaches have significantly matured in the last few years and have greatly facilitated investigations of host proteome during *T*. *gondii* infection. For example, proteome of *T*. *gondii*-infected macrophage from Kunming mice 24 hour post infection exhibited altered expression of 38 proteins involved in metabolism, cell structure, signal transduction and immune response [14]. Similar proteomic profile was obtained from brain tissues of Kunming mice infected with genotype II *T*. *gondii* PRU strain [15]. Also, proteome of human foreskin fibroblasts (HFFs) infected with genotype II *T*. *gondii* RH strain revealed differentially expressed proteins involved in many metabolic processes including glycolysis, lipid metabolism, cell death, stress, and immune response [13]. As can be gleamed from the wealth of proteomics-based information available, this approach has contributed greatly to our understanding of molecular mechanisms underlying host response to *T*. *gondii* infection and clearly indicates global reprogramming of host cell metabolism by the parasite. However, previous studies have been based on two-dimensional sodium dodecyl sulfate polyacrylamide gel electrophoresis [13–15], which is relatively low throughput for protein identification or quantification. Also, there are no papers reporting the use of iTRAQ-based proteomics to assay mouse liver samples for hepatic toxoplasmosis investigation. Animal models have been extremely helpful for providing fundamental insights into the pathogenesis of *T*. *gondii* infection. While some of the features of specific models may not fully mimic human pathophysiological changes, animal models provide a system to understand some of the basic host responses to infection. The aim of the present study was to investigate the global proteomic changes in the liver of mice infected with *T*. *gondii*. The unbiased iTRAQ proteomic method was employed to understand mechanisms of liver injury that occur during acute infection with *T*. *gondii*.

## Materials and Methods

### Ethical statement

All animal procedures were approved by the Animal Ethics Committee of Lanzhou Veterinary Research Institute, Chinese Academy of Agricultural Sciences (Permit No. LVRIAEC2013-006). All experiments were performed in strict compliance with requirements of the Animal Ethics Procedures and Guidelines of the People's Republic of China.

### Mice, parasite challenge and sample collection

The PYS strain of *T*. *gondii* (genotype ToxoDB#9) the most widespread genotype in China [13], was isolated and maintained in our laboratory. BALB/c mice were purchased from the Laboratory Animal Center of Lanzhou Veterinary Research Institute, Chinese Academy of Agriculture Science. The health status and behavior of mice were examined daily. Mice had *ad libitum* access to food and water. Six-week-old female BALB/c mice were randomly divided into two equal groups (3 mice each group). Each mouse of the infected group was injected intraperitoneally (i.p.) with 200 tachyzoites of the PYS strain counted by hemocytometer, whereas mice in the control group were mock-inoculated with phosphate buffer saline (PBS, pH7.4) without tachyzoites. Six days post-infection (PI) mice were humanely sacrificed by CO2 asphyxiation. The liver tissues were collected and stored at -80°C until use. *T*. *gondii* infection was confirmed by microscopic examination and PCR of liver DNA as described previously [20]. PCR positive products were submitted to Genewiz Company (Beijing, China) for sequencing using ABI3730 sequencer on both strands.

### Protein extraction

Total protein from the each cryo-preserved mouse liver samples was prepared as follows. Each biological sample was individually ground to powder in liquid nitrogen and extracted in Lysis buffer 1 (7 M Urea, 2 M Thiourea, 4% CHAPS, 40 mM Tris-HCl, pH 8.5) containing 1 mM PMSF and 2 mM EDTA (final concentration). After 5 min, 10 mM DTT (final concentration) was added to the sample. The suspension was sonicated at 200 W for 15 min and then centrifuged at 4°C, 30,000 *g* for 15 min. The supernatant was mixed with 5 volumes of chilled acetone containing 10% (v/v) TCA and incubated at -20°C overnight. After centrifugation at 4°C, 30,000 *g* for 15 min, the supernatant was discarded. Then, the precipitate was washed with chilled acetone three times. The pellet was air-dried and dissolved in Lysis buffer 2 (7 M urea, 2 M thiourea, 4% NP40, 20 mM Tris-HCl, pH 8.0–8.5). The suspension was sonicated at 200 W for 15 min and centrifuged at 4°C, 30,000 *g* for 15 min. The supernatant was then transferred to another tube. To reduce disulfide bonds in proteins of the supernatant, 10 mM DTT (final concentration) was added and incubated at 56°C for 1 hour. Subsequently, 55 mM IAM (final concentration) was added followed by incubation for 1 hour in the dark. The supernatant was mixed with 55 volumes of chilled acetone for 2 hour at -20°C to precipitate proteins. After centrifugation at 4°C, 30,000 *g* for 15 min, the supernatant was discarded and the pellet was air-dried for 5 min, dissolved in 500 μl 0.5 M TEAB (Applied Biosystems, Milan, Italy), and sonicated at 200 W for 15 min. Finally, samples were centrifuged at 4°C, 30,000 *g* for 15 min. The supernatant was transferred to a new tube and proteins were quantified, using the Bradford method [21]. The proteins in the supernatant were stored at -80°C until further analysis.

### iTRAQ labeling and SCX fractionation

Proteins (100 μg) from each sample were digested with Trypsin Gold (Promega, Madison, WI, USA) with the ratio of protein:trypsin = 30:1 at 37°C for 16 hour. After trypsin digestion, peptides were dried by vacuum centrifugation and were reconstituted in 0.5 M TEAB and processed according to the manufacture’s protocol for 8-plex iTRAQ reagent (Applied Biosystems).

SCX chromatography was performed with an LC-20AB HPLC Pump system (Shimadzu, Kyoto, Japan). The iTRAQ-labeled peptide mixtures were reconstituted with 4 ml buffer A (25 mM NaH_2_PO_4_ in 25% ACN, pH 2.7) and loaded onto a 4.6×250 mm Ultremex SCX column containing 5 μm particles (Phenomenex). The peptides were eluted at a flow rate of 1 ml/min with a gradient of buffer A for 10 min, 5–60% buffer B (25 mM NaH_2_PO_4_, 1 M KCl in 25% ACN, pH 2.7) for 27 min, 60–100% buffer B for 1 min. The system was then maintained at 100% buffer B for 1 min before equilibrating with buffer A for 10 min prior to the next injection. Elution was monitored by measuring the absorbance at 214 nm, and fractions were collected every 1 min. The eluted peptides were pooled into 20 fractions, desalted with a Strata X C18 column (Phenomenex) and vacuum-dried.

### LC-ESI-MS/MS analysis based on Triple TOF 5600

Each fraction was resuspended in buffer A (5% ACN, 0.1% FA) and centrifuged at 20,000 *g* for 10 min, and the final concentration of peptide was 0.5 μg/μl. About 10 μl supernatant was loaded on a LC-20AD nanoHPLC (Shimadzu, Kyoto, Japan) by the auto sampler onto a 2 cm C18 trap column. Then, the peptides were eluted onto a 10 cm analytical C18 column (inner diameter 75 μm) packed in-house. The samples were loaded at 8 μL/min for 4 min, then the 35 min gradient was run at 300 nl/min starting from 2 and increasing to 35% buffer B (95% ACN, 0.1% FA), followed by 5 min linear gradient to 60%, followed by 2min linear gradient to 80%, and maintenance at 80% buffer B for 4 min, and finally a return to 5% for 1 min.

Data acquisition was performed with a TripleTOF 5600 System (AB SCIEX, Concord, ON) fitted with a Nanospray III source (AB SCIEX, Concord, ON) and a pulled quartz tip as the emitter (New Objectives, Woburn, MA). Data was acquired using an ion spray voltage of 2.5 kV, curtain gas of 30 psi, nebulizer gas of 15 psi, and an interface heater temperature of 150°C. The MS was operated with a RP of greater than or equal to 30,000 FWHM for TOF MS scans. For IDA, survey scans were acquired in 250 ms and as many as 30 product ion scans were collected if exceeding a threshold of 120 counts per second (counts/s) and with a 2+ to 5+ charge-state. Total cycle time was fixed to 3.3 s. Q2 transmission window was 100 Da for 100%. Four time bins were summed for each scan at a pulser frequency value of 11 kHz through monitoring of the 40 GHz multichannel TDC detector with four-anode channel detect ion. A sweeping collision energy setting of 35±5 eV coupled with iTRAQ adjust rolling collision energy was applied to all precursor ions for collision-induced dissociation. Dynamic exclusion was set for 1/2 of peak width (15 s), and then the precursor was refreshed off the exclusion list.

### Proteomic data analysis

Discoverer 1.2 (PD 1.2, Thermo), 5600 msconverter and the MGF file were searched. Protein identification was performed by using the Mascot search engine (Matrix Science, London, UK; version 2.3.02) against the Uniprot database. For protein identification and quantitation, a protein was required to contain at least two unique peptides. The peptide for quantification was automatically selected by the algorithm to calculate the reporter peak area, error factor (EF) and *p*-value (default parameters in Mascot Software package). Student’s t-test was performed using the Mascot software. The resulting data set was auto bias-corrected to the biological replicate. The quantitative protein ratios were weighted and normalized by the median ratio in Mascot. We only used ratios with *p* values < 0.05, and only fold change >1.5 was considered as significant.

Functional enrichment of the differentially expressed proteins (DEPs) or cellular organelle components was conducted using a cytoscape plugin, BiNGO [22], and all data files used in BiNGO were downloaded from the Gene Ontology database (GO, http://www.geneontology.org/) [23]. Pathway analyses of DEPs were performed through the KEGG database (KEGG, http://www.genome.jp/kegg/) [24]. Hypergeometric test and the Benjamini-Hocherg False Discovery Rate (FDR) correction were used for identifying significantly enriched GO terms or KEGG pathways. Significance levels of *p* < 0.05, following FDR correction, were used as cut-offs for the enrichment threshold of GO term or KEGG pathway. Differently expressed GO terms or KEGG pathways were also identified where the FDR corrected *p* value was less than 0.05, the number of DEPs was greater than 3, and the ratio between upregulated protein and downregulated protein was greater than 2 or less than 0.5. The relationships among metabolism of substances, liver damage and DEPs were analyzed using the Comparative Toxicogenomics Database (http://ctdbase.org/) [25]. Transcription factor analysis of DEPs was analyzed by Pscan web service (http://159.149.160.51/pscan/) [26]. The mass spectrometry proteomics data have been deposited at the ProteomeXchange Consortium [27] via the PRIDE partner repository with the dataset identifier PXD003399.

## Results

### Confirmation of *T*. *gondii* infection in mouse livers

All 3 biological replicates in the infected group exhibited signs indicative of toxoplasmosis, but no mouse has died of *T*. *gondii* infection. Also, *T*. *gondii* tachyzoites were observed in peritoneal fluid of all infected mice. In contrast, no clinical signs or tachyzoites of *T*. *gondii* were observed in the non-infected control mice. Liver samples from all infected mice were PCR-positive and DNA sequencing confirmed that all PCR products are amplicons of B1 gene of *T*. *gondii*. In contrast, the negative PCR control template and all DNA templates of the control mouse livers were PCR negative.

### Proteomic characterization

**Fig 1:**
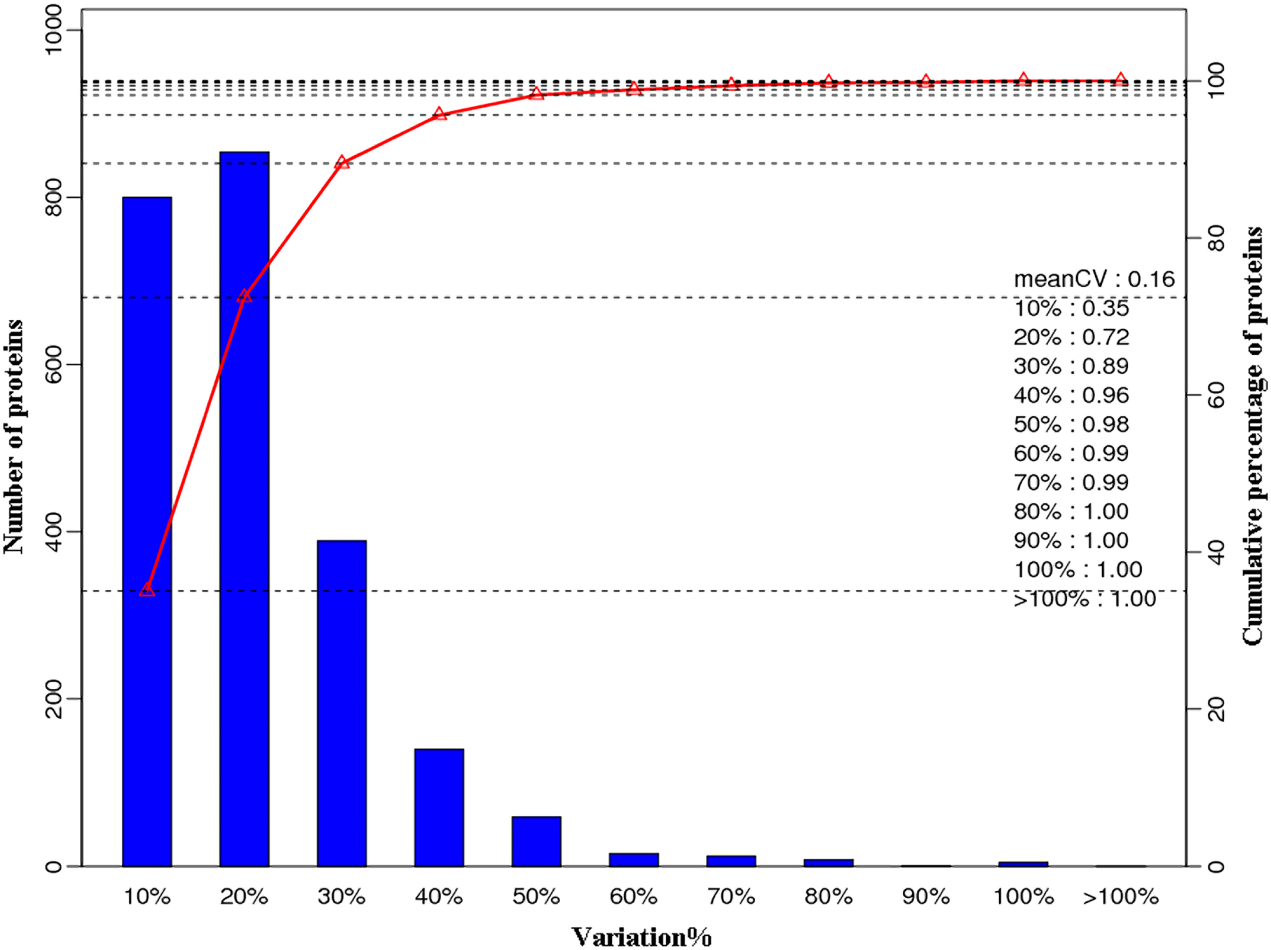
Coefficients of variation values between the three biological replicates. Bottom-axis (%variation) shows the variation of identified proteins. Left-axis shows the number of identified proteins. Right-axis and red line show the cumulative percentage of identified proteins.

A total of 407,280 spectra were obtained. According to the Mascot program searching, 46,681 spectra were matched and 15,699 high scoring unique peptides were obtained. A total of 3,643 proteins were identified, 301 of which were differentially expressed in infected livers (*p* < 0.05), including 73 downregulated proteins and 228 upregulated proteins. The details of differentially expressed proteins are shown in S1 Table. The variation coefficients between three biological replicates are shown in Fig 1. Variability of 72% protein was not more than 20%.

**Fig 2:**
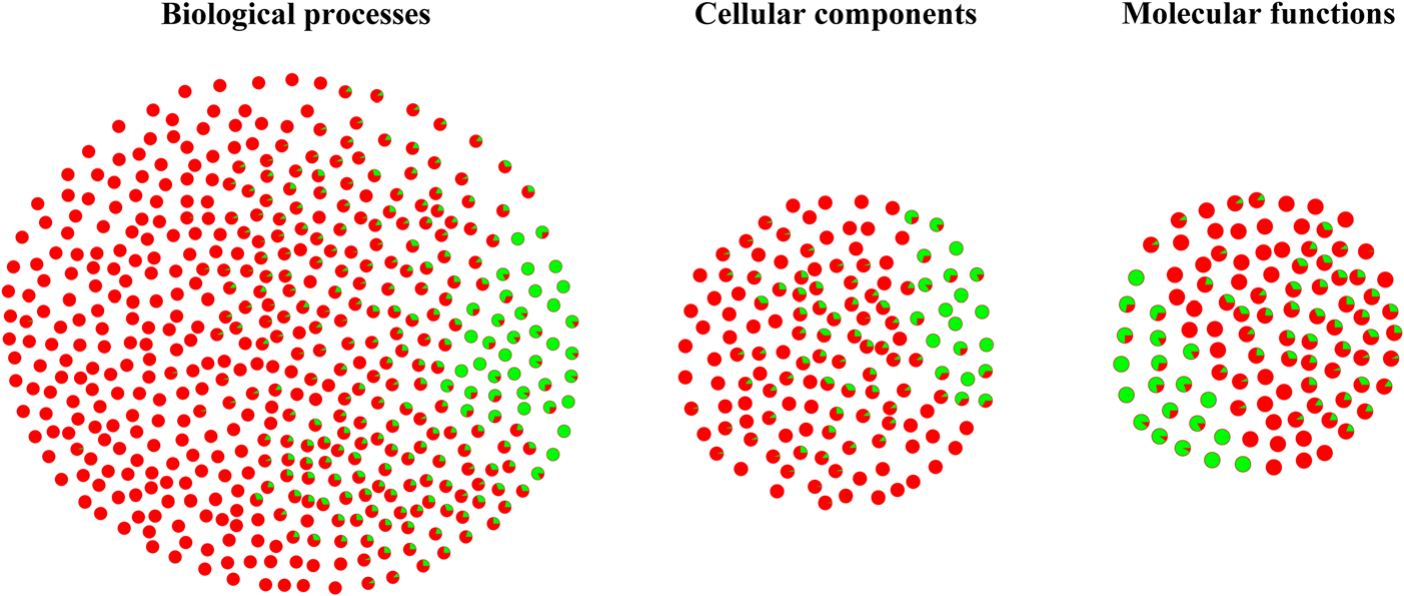
Differentially expressed GO term of infected liver. Pie dot shows the differentially expressed GO terms. Red color represents the percentage of upregulated proteins. Green color represents the percentage of downregulated proteins.

**Fig 3:**
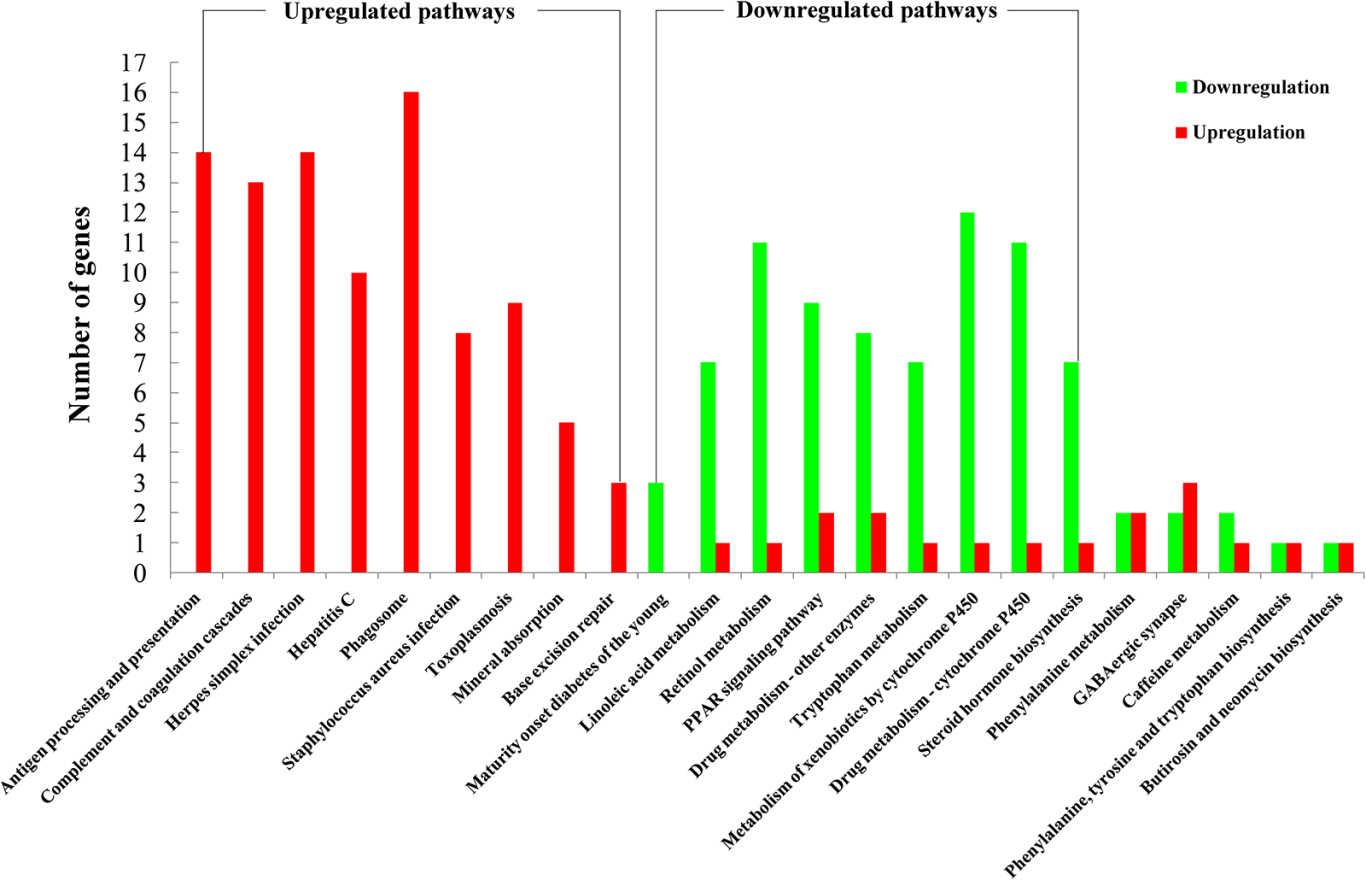
Enriched pathways in KEGG database. 23 pathways were enriched in the KEGG database. Red color represents upregulation. Green color represents downregulation.

GO analysis was performed to study the biological change of infected liver. 139 cellular components, 103 molecular functions and 473 biological processes of infected liver were differentially expressed in the present study. As shown in Fig 2, most differentially expressed GO terms were upregulated, including cellular components (vesicle, nucleus, Golgi apparatus, lysosome, endosome, actin cytoskeleton) and biological processes (material transport, actin cytoskeleton organization, immune response related terms, protein complex assembly, protein folding, proteolysis, negative regulation of cell death and nucleobase-containing small molecule metabolic process). However, downregulated GO terms were also detected, such as cellular components (mitochondrial envelope, microbody), biological processes (alpha-amino acid catabolic process, oxidation-reduction process, organic acid metabolic process, fatty acid metabolic process, cellular lipid metabolic process, drug or xenobiotic metabolic process) and molecular oxidoreductase activity and heme binding. The details of differentially expressed proteins of cellular components, biological processes and molecular functions are listed in S2–S4 Tables, respectively. As shown in Fig 3, 23 pathways were significantly enriched in KEGG analysis, including 9 upregulated pathways and 9 downregulated pathways. Consistent with GO enrichment analysis, most upregulated pathways were involved in immune response, while downregulated pathways were metabolism-related.

### Comparative toxicogenomics analysis

In the present study, we identified 15 DEPs involved in drug or xenobiotic metabolism, including Gstm1, Sult2a5, Sult2a1, Cyp2c54, Cyp3a16, Cyp3a11, Cyp8b1, Cyp2c50, Cyp2b9, Cyp4a10, Cyp3a25, Cyp1a2, Cyp2a22, Cyp2c29 and Cyp3a13. Among the DEPs, only Cyp3a13 was upregulated in mouse livers infected with *T*. *gondii*. According to the Comparative Toxicogenomics Database, over 400 substances were catabolized by the DEPs described above. The details of relationships between the DEPs and chemicals are listed in S5 Table. We also analyzed all DEPs in the Comparative Toxicogenomics Database to obtain a list of liver diseases that correlate with DEPs. Cancer, liver cirrhosis, drug-induced liver injury, cholestasis, drug eruptions, fatty liver, inflammation and necrosis were shown in the results of the Comparative Toxicogenomics Database analysis.

### Transcription factor analysis of DEPs

In the present study, 2 transcription factors (Ybx1, Stat3) and 6 transcription co-factors (Calr, Ewsr1, Pml, Mybbp1a, Thrap3 and Ddx17) were upregulated. In Pscan analysis, 263 transcription factor motifs were employed to analyze the transcription factors that could be used by DEPs. The top 10 transcription factors used by the upregulated proteins were Stat2, Stat1, Irf2, Irf1, Sp2, Egr1, Stat3, Klf4, Elf1 and Gabpa, while the top 10 transcription factors used by the downregulated proteins were Hnf4A, Ewsr1, Fli1, Hnf4g, Nr2f1, Pparg, Rxra, Hnf1A, Foxa1 and Foxo1.

## Discussion

Over the last decade, a significant amount of data, including serological, biochemical and direct detection studies, has suggested a potential role for *T*. *gondii* infection in liver pathologies [3–6,8–11]. However, the mechanisms involved in establishing hepatic infection with this organism remain unclear.

Unbiased iTRAQ-based proteomic analysis revealed 301 differentially expressed proteins in mouse livers and most of them were upregulated. These altered proteins play crucial roles in several cellular functions, particularly in transcription and translation processes. Our results demonstrate that host protein involved in cellular immune defense, lipid metabolism, and intracellular transport are regulated during the early stage of *T*. *gondii* infection. Potential roles of the altered proteins in response to acute *T*. *gondii* infection in the present study corroborated the current knowledge of *T*. *gondii* infection. *T*. *gondii* has developed strategies to manipulate important host cell immune pathways. Indeed, infection of mammalian cells with *T*. *gondii* induces many changes in host cell gene transcription, including those genes involved in energy metabolism, immune responses and signaling [28]. Low variability among the tested biological replicates indicates consistency in our results. GO and KEGG enrichment analyses were applied to study the biological change of mouse livers infected by *T*. *gondii*. Several GO terms were downregulated, such as the components of mitochondrial envelope, microbody, oxidation-reduction process, organic acid metabolic process, fatty acid metabolic process, cellular lipid metabolic process, drug or xenobiotic metabolic process, oxidoreductase activity and heme binding. On the other hand, the upregulation of GO terms was also detected, such as vesicle, Golgi apparatus, lysosome, endosome, material transport, actin cytoskeleton, protein complex assembly, protein folding, proteolysis, negative regulation of cell death and immune response-related terms. To the best of our knowledge, the present study is the first to deploy iTRAQ-based proteomic method to address changes in the cellular proteome of mouse liver during *T*. *gondii* infection. Our findings will be discussed in the light of how the parasite alters the immune response, as this is crucial for our understanding of how the infection is controlled, and as discussed below, the host immune response is a major target of *T*. *gondii* manipulation.

### Immune response

Activation of a cell-mediated immunity plays a crucial role in protection and pathogenesis of *T*. *gondii* infection [29]. Stimulation of host cells by IFN-γ inhibits growth of *T*. *gondii* primarily by induction of indoleamine 2,3-dioxygenase (IDO) activity, which deprives the parasite of the essential amino acid tryptophan. Also, *T*. *gondii* upregulates inducible nitric oxide synthase (iNOS) to diminish arginine levels and upregulates interferon-inducible immunity related GTPases (IRGs) and guanylate binding proteins (GBPs) to disrupt the parasitophorous vacuole membrane (PVM), which separates the parasite from host immune defences [30]. IFN-γ was not detected in the present study, probably due to the limitation of the mass-spectrometric technique (i.e. at least two unique peptides are required for the identification of a specific protein and mass-spectrometry cannot identify or distinguish all proteins in a biological system [31]). However, five immunity-related GTPases (IRGs) (Gm12250, Igtp, Iigp1, Irgm1, Gm495) and five GBPs (Gbp7, Gbp6, Gbp4, Gbp2b, Gbp2) were upregulated DEPs. The roles of Irgm1, Iigp1, Igtp, Gm12250 (also known as Irgb10), Gbp7, Gbp6, Gbp2 and Gbp4 in the disruption of PVM of *T*. *gondii* have been described [32–36]. Interestingly, one indoleamine 2,3-dioxygenase (Ido2) was downregulated in the infected livers. This downregulation may have a protective role for *T*. *gondii* by preventing the decrease of host tryptophan levels. IFN-γ can be produced by IL-1β pathway activation [37]. However, in the present study, the IL-1β pathway was inhibited by the upregulation of the interleukin 1 receptor antagonist (Il1rn), which blocks the IL-1β signal [38]. Upregulation of Il1rn was not reported in previous proteomic reports [13–15]. The discrepancy between our results and the reported proteomic findings may be attributed to the use of low throughput proteomic method (e.g., DIGE) [13–15]. Regardless, the upregulation of immune response-related terms is, overall, consistent with previous proteomic reports in previous studies [13–15].

### Regulatory elements and signaling pathways

Because protein expression is controlled by *Cis*-regulatory elements and many innate immune effectors are under the control of transcription factors in the anti-*T*. *gondii* immune response, we performed *Cis*-regulatory element analysis to obtain more insights into the altered biological processes of the infected liver. The top 10 transcription factors involved in regulation of upregulated genes were Stat2, Stat1, Irf2, Irf1, Sp2, Egr1, Stat3, Klf4, Elf1 and Gabpa, while the top 10 transcription factors used by downregulated genes were Hnf4A, Ewsr1, Fli1, Hnf4g, Nr2f1, Foxa1, Foxo1, Hnf1A, Pparg and Rxra.

Infection with *T*. *gondii* interferes with host cell signaling pathways implicated in protective immunity, for instance by blocking the transcription factors signal transducer and activator of transcription 1 (STAT1) [39] and nuclear factor kappa-light-chain-enhancer of activated B cells (NF-κB) [40]. Fibroblasts infected with any of the three clonal types of *T*. *gondii* (i.e. I, II and III) are unresponsive to the anti-parasitic effects of IFN-γ and this appears to be due to impaired STAT1 signaling [41]. In *T*. *gondii*-infected bone marrow derived macrophages, this occurs due to modifications in chromatin structure, which disrupt STAT1 binding to nuclear promoters [42]. Also, *T*. *gondii* infection upregulates anti-inflammatory pathways including those involving STAT3 [43] and the suppressor of cytokine signaling protein (SOCS) 1 [44], SOCS3 [45], potentially compromising host resistance mechanisms to *T*. *gondii* infection.

Two important transcription factors Pparg and Rxra were involved in the regulation of downregulated proteins. According to the KEGG database, Pparg (also known as Nr1c3) and Rxra (also known as Nr2b1) are critical transcription factors of the peroxisome proliferator-activated receptor (PPAR) signaling pathway, which play a key role in modulating hepatic triglyceride accumulation in liver. In our study, PPAR signaling pathway was significantly enriched (Fig 3), and 9 differentially expressed proteins, such as Ehhadh, Acsl1, Acaa1b, Cyp8b1, Me1, Apoa2, Apoc3, Cyp4a10, and Fads2 involved in PPAR signaling pathway were downregulated. These findings show that *T*. *gondii* may regulate fatty acid metabolism via regulating PPAR signaling pathway to downregulate fatty acid metabolism. Differential regulation of a variety of lipid species has also been reported previously [13].

### Cell structure

The host immune responses do not operate in isolation. In fact, anti-*T*. *gondii* immune response is engaged with many cellular components and biological processes, such as cellular organelles, protein folding, protein complex assembly, proteolysis, material transport and host secretion or endocytosis. Most GO terms of these were upregulated as listed in S2–S4 Tables. Secretion, endocytosis, and phagocytosis are controlled by vesicle-mediated transport processes, which mediate exchange of materials among different cellular organelles, such as the endoplasmic reticulum (ER), Golgi apparatus, lysosome, endosome and other cellular organelles. Most importantly, this process sorts and transports proteins to where they are functionally required [46,47]. For example, MHC complexes are sorted and transported by vesicle-mediated transportation after synthesis and assembly [47]. In the present study, vesicle-mediated transport processes were upregulated. However, at mRNA level, Tanaka *et al*. reported that vesicle-mediated transport process was downregulated in mouse brains infected by *T*. *godnii* genotype I strain [48]. This inconsistency may be related to the use of different host tissues, different *T*. *gondii* strains or experimental conditions.

### Lipid and energy metabolism

**Fig 4:**
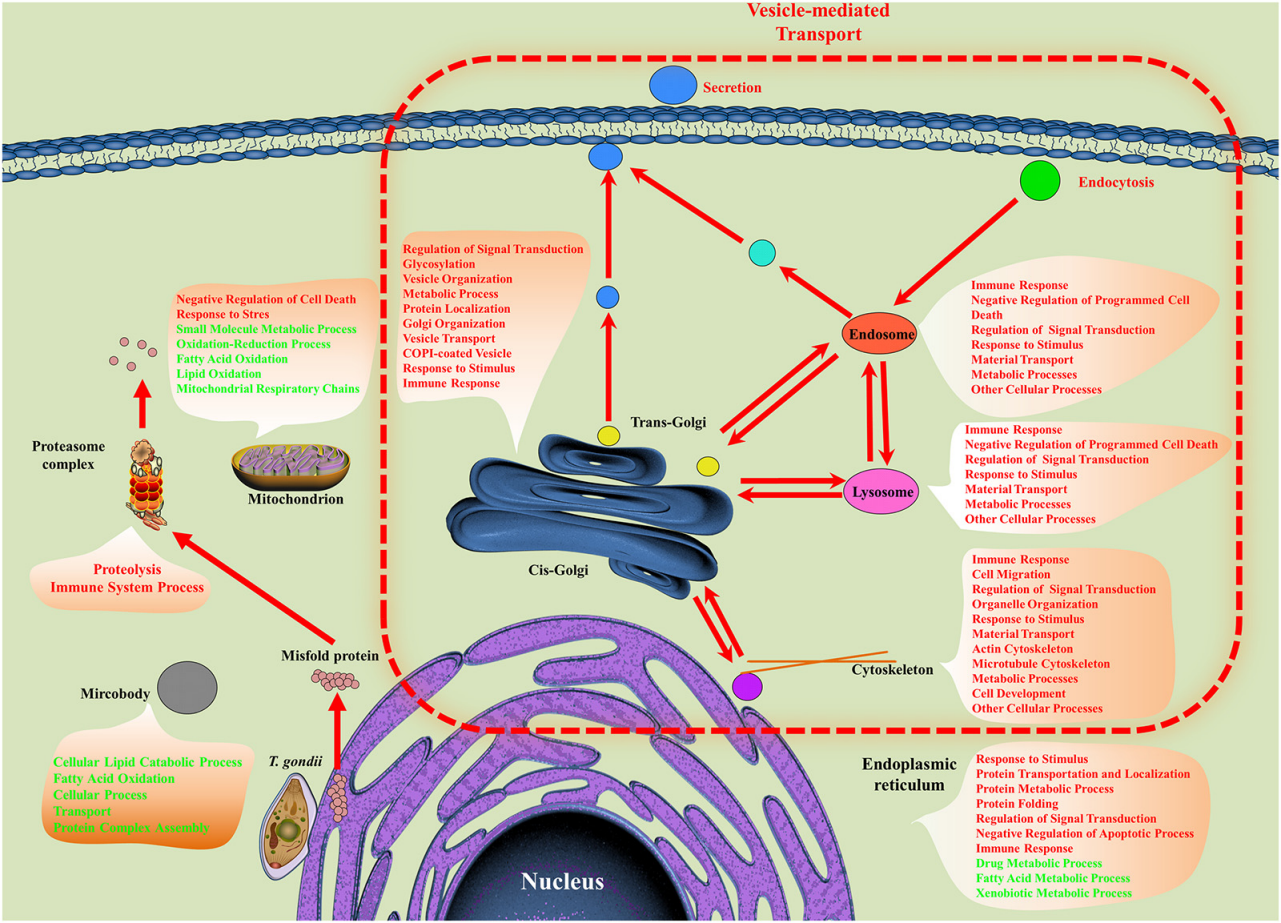
Differentially expressed GO term of cellular organelle. The processes, such as secretion, endocytosis and vesicle-mediated transport were enriched by all DEPs. GO term of cellular organelles were enriched using differentially expressed cellular organelle components. All enrichment analysis was performed on BiNGO plugin. Red color represents upregulated GO term. Green color represents downregulated GO term.

As shown in Fig 4, some biological processes or functions of hepatocellular organelles were altered. For example, negative regulation of cell death was upregulated in lysosomes, endosomes, mitochondria and ER, which is consistent with the anti-apoptotic activity of *T*. *gondii* [49]. In the mitochondria of hepatocytes, energy production was decreased by downregulation of mitochondrial respiratory chain, lipid oxidation, and fatty acid oxidation. Lipid catabolic processes and fatty acid oxidation were downregulated in microbodies too. Mitochondria, lipids or fatty acids are important sources of energy, which is needed for many biological processes. These downregulations imply that hepatocellular energy metabolism was affected by *T*. *gondii* infection. Severe alterations of energy production in mitochondria could result in several liver diseases such as liver necrosis, steatosis or steatohepatitis [50]. These diseases are predicted to be linked to the DEPs obtained in the present study as indicated by the Comparative Toxicogenomics Database analysis.

### Drug detoxification

The liver is the major detoxifying organ in the mammalian body. Its ability to clear most drugs and other xenobiotics determine the efficacy and toxicity associated with any given drug. Previous reports showed that *T*. *gondii* infection can alter drug pharmacokinetics too [10,11]. In general, xenobiotic or drugs metabolism *in vivo* occurs in three phases [51]. Phase-I is mainly mediated by the members of cytochrome P450 family which transform lipophilic materials into soluble hydrophilic substrates. The products of phase-I are toxic or electrophilic intermediates and if these products not transformed into nontoxic substances by the metabolic process of phase-II, cancer or tissue damage could be induced [52]. The phase-II reactions are mediated by several transferases or oxidoreductase, such as UDP glucuronyltransferases, glutathione-S-transferases, sulfotransferases, alcohol dehydrogenase, aldo-keto reductase, flavin containing monooxygenase and other enzymes involved in xenobiotic or drug metabolic processes. In phase-III, all products that are detoxified by phase-II reactions are excreted into bile via export pumps and can then be eliminated from the body. In the present study, one glutathione S-transferase (Gstm1), two sulfotransferases (Sult2a1 and Sult2a5), eleven cytochrome P450 (Cyp2c54, Cyp3a16, Cyp3a11, Cyp8b1, Cyp2c50, Cyp2b9, Cyp4a10, Cyp3a25, Cyp1a2, Cyp2a22, and Cyp2c29) and one bile salt export pump (Abcb11) were downregulated. Only one cytochrome P450 (Cyp3a13) was upregulated in infected liver.

Downregulated metabolisms of macrolide antibiotics and cyclosporin A, which are listed in S5 Table, have been observed during *T*. *gondii* infection [10,11]. Previous reports showed that the altered pharmacokinetics of macrolide antibiotics or cyclosporin could result in severe adverse drug reactions [53,54]. Therefore, knowledge of the pharmacokinetics changes that occur in toxoplasmosis is very important for drug prescription purposes particularly when multiple drugs or doses are taken in conjunction. According to the analysis of the Comparative Toxicogenomics Database, the metabolism of over 400 substances were altered in infected liver, including that of antibiotics, pesticides, tranquilizer, glucocorticoids, and other xenobioticsor/drugs. The top 10 substances that their metabolism could be affected by *T*. *gondii* infection were 1,4-bis[2-(3,5-dichloropyridyloxy)]benzene, dietary fats, phenobarbital, perfluorooctanoic acid, pirinixic acid, benzo(a)pyrene, diethylnitrosamine, tetrachlorodibenzodioxin, clofibrate, and acetaminophen. Acetaminophen (also known as N-acetyl-p-aminophenol or paracetamol) is a commonly used analgesic and antipyretic drug that can cause extensive hepatic damage after an excessive dose and is the leading cause of drug-induced liver injury in the USA [55]. Acetaminophen-mediated hepatotoxicity has been thought to be attributed to an initial oxidative stress followed by energetic stress, mitochondrial dysfunction and upregulation of glycolysis [56]. Similar metabolic alterations have been observed during *T*. *gondii* infection [57]. Therefore, failure to take these changes into consideration when acetaminophen is prescribed to toxoplasmosis patients can result in increased risk of developing severe acetaminophen-induced acute liver damage. *T*. *gondii* infection will not only potentiate the oxidative and bioenergetic stress caused by acetaminophen, but will also change the drug pharmacokinetic via downregulation of the drug metabolic processes It is possible that the xenobiotic or drugs metabolics changes are not specific to *T*. *gondii* infection. However, our data clearly indicate that proteomic alterations are not global, but rather target specific cellular functions, such as the metabolism of the xenobiotic/drugs metabolism in mouse liver following acute *T*. *gondii* infection. Also, the mouse model and approach used in our study can be useful in elucidating the pharmacokinetic changes of any given compound before being prescribed to toxoplasmosis patients.

In summary, we profiled, for the first time, the mouse liver proteome following acute *T*. *gondii* infection at a global level. Our findings demonstrate that *T*. *gondii* infection has wide spread effects on the expression of host proteins involved in host immune defense mechanisms, lipid metabolism, intracellular transport, and drug pharmacokinetics. The results of this research lead to an important question: could the interaction of *T*. *gondii* with liver tissue impair mitochondrial functions and liver metabolism to such a level that *T*. *gondii*-induced hepatic dysfunction might be explained? There is evidence that *T*. *gondii* infection is associated with extensive alterations in the liver of affected hosts [3–6,8–11]. The biomarker molecules and pathways identified in our study also provide insights into some of the underlying causes of *T*. *gondii*-associated liver pathology. Inefficient mitochondrial metabolism can affect hepatocellular energy metabolism and could result in several liver diseases such as liver necrosis, steatosis or steatohepatitis [50], highlighting the importance of further efforts to understand the interaction of *T*. *gondii* with mitochondria and its influence on hepatic metabolism. While hundreds of differentially expressed proteins are observed in our study, the validation and clinical value of these proteins warrants further investigation.

## Supporting Information

S1 Table Differentially expressed proteins. (XLSX)

S2 Table Differentially expressed cellular components of GO enrichment. (XLSX)

S3 Table Differentially expressed biological processes of GO enrichment. (XLSX)

S4 Table Differentially expressed molecular functions of GO enrichment. (XLSX)

S5 Table Interaction between DEPs of xenobiotic metabolic process and chemicals. (XLSX)
